# “I want one nurse who is friendly to talk to me properly like a friend”: Learner preferences for HIV and contraceptive service provision in Gauteng, South Africa

**DOI:** 10.21203/rs.3.rs-3725260/v1

**Published:** 2023-12-12

**Authors:** Aneesa Moolla, Mongwenyana Constance, Nkosinathi Ngcobo, Sithabile Mngadi, Caroline Govathson, Lawrence Long, Sophie Jane Pascoe

**Affiliations:** University of the Witwatersrand; University of the Witwatersrand; University of the Witwatersrand; University of the Witwatersrand; University of the Witwatersrand; Boston University; University of the Witwatersrand

**Keywords:** Focus Group, Interview, Adolescent, Schools, Sexual and reproductive health

## Abstract

**Background:**

Research with adolescents indicates that youth aged 15–24 years, especially females, are at high risk for HIV infection. The overall HIV prevalence among youth in this age group was 6.2% estimated in 2022. In addition, > 800,000 adolescents are newly infected with HIV every year and 79% of these infections occur in sub-Saharan Africa. The health service provision preferences and needs of adolescents are critical to reaching this population.

**Methods:**

This qualitative study was conducted with learners from three public secondary schools in Gauteng, South Africa. Using convenience sampling, 22 in-depth stakeholder interviews (KIIs) with stakeholders and 8 focus group discussions (FGDs) with 55 learners aged ≥ 15, were conducted between March and October 2018. Learners < 18 were given assent and parental consent forms, whilst those ≥ 18 could consent of their own accord. KIIs and FGDs were conducted in private venues in the preferred language by trained interviewers and audio-recorded. Audio files were transcribed verbatim and translated into English if needed. Data were analysed thematically using NVivo version 11.

**Results:**

The findings from both stakeholders and learners indicate many critical accessibility barriers which include: negative healthcare staff attitudes from older judgemental staff; stigmatisation from healthcare workers, the community as well as family; a lack of private consulting spaces and no confidentiality of patient information at facilities; inconvenient clinic operating times; long queues and facility resource issues. Both groups of participants suggested that accessibility to healthcare could be improved through value-added services (including free Wi-Fi and food), social gatherings and educational information sessions, as well as being staffed by younger, friendlier, confidential and non-judgemental staff in a private healthcare setting.

**Conclusion:**

It is clear that there are many critical barriers that deter learners from accessing HIV and contraceptive services. Provision of private rooms and trying to ensure information confidentiality for youth-friendly services at locations and times that can be easily accessed by learners is key. Greater emphasis on learner-parent-teacher communication around sexual health education at school is needed along with making this information being more readily available to learners.

## Background

Adolescence is a difficult and critical time of experimentation, new experiences and vulnerability. Experimentation may include drugs, reflecting on sexual orientation and also sexual experiences [1]. Globally, youth risk behaviour, including risky sexual behaviour, drug abuse and violence, is a public health concern and South Africa is no exception [2]. Estimates show that approximately 14 million young people die each year from Sexual and Reproductive Health (SRH) challenges, despite availability of social, economic, demographic and health benefits of safe sex [3]. The SSA region accounts for the highest burden of adolescent HIV globally, and adolescent girls, in particular, are disproportionately affected, accounting for three out of four new HIV infections [4].

The latest UNAIDS statistics show that adolescent girls and young women (AGYW) from sub-Saharan Africa [5], aged 15–24, remain at substantial risk of acquiring HIV [5]. Every week, an estimated 4900 incident infections occur among women in this age group globally [5]. In SSA, approximately six out of seven new infections occur among adolescents aged 15–19 years, and young women aged 15–24 years are twice likely to be living with HIV than their male counterparts [5]. AGYW accounted for 63% of all new HIV infections in 2021 [5]. Expedited prioritization and actions to reduce incident infections in particular among adolescent girls and young women from sub-Saharan Africa are therefore required to attain the UNAIDS 95–95-95 targets by 2030 [5]. The distribution of STIs, as well as HIV incidence and prevalence in heterosexuals, is shifted toward younger age groups especially for females in comparison to males [6]. In SSA age disparity is positively associated with HIV prevalence where the incidence of HIV increases swiftly from young to middle age [6].

For older adolescents aged 15–19 years, knowledge of HIV and other sexually transmitted diseases is important to promote the use of condoms and prevent sexually transmitted disease transmission and unintended pregnancy [7]. For younger adolescents, knowledge of reproductive health (menstruation, for example) and the implications of this for sex and reproduction are important in understanding the consequences of sexual debut [7]. The National Adolescent and Youth Indicators report revealed that most study participants had the correct knowledge, in that they knew condoms are effective at preventing the spread of HIV; but also reported a high teenage pregnancies and termination of pregnancies amongst girls aged 10–19 years in Limpopo province [8].

In many sub-Saharan Africa countries, sexual and reproductive health (SRH) needs of young people / youth are often underserved and underestimated despite their demonstrated need and the urgency of these services [9]. This is partly explained by multiple barriers in accessing SRH services including inconvenient operating times, long distance and waiting times, parental consent, transport costs, fears about interactions with healthcare staff which includes fear of judgemental staff and the lack of both confidentiality relating to non-divulging of personal patient information by staff, as well as privacy at clinics which relates to lack of secluded patient consulting stations [10]. There is also considerable stigma associated with premarital sexual activity in many South African communities, including being shamed by health care workers and this is linked to low levels of service utilization [11]. This has led youth into risky sexual behaviour resulting in high STI and HIV prevalence among young people, early pregnancy, and vulnerability to unsafe abortions and delivery complications resulting in high rates of death and disability [9], [12], [13].

In terms of services, evidence shows that school health programs can support young people in adopting lifelong behaviours and attitudes that reduce their risk of HIV, other STIs, and pregnancies [14], [15],[16]. Based on this, the South African government has subsequently taken steps to put an Integrated School Health Program (ISHP) in place [8] but evidence of successes and challenges are not currently available as these projects often have limited coverage or limited periods of implementation or follow-up [9]. For any school-based program to be deemed successful, learners have to use the services that are provided. The program must also be comprehensively tailored to meet the needs of the population it intends to serve. Thus, seeking out adolescents’ views and preferences for HIV and contraceptive services will highlight both promising directions and persistent challenges in preventing pregnancies and treating HIV and other STIs in this population.

Though significant progress has been made in advocating for the rights of adolescents to access HIV and contraceptive services; many young people in Sub-Saharan African still face the risk of HIV, STIs and unintended pregnancies [17], [18], [19], [20]. This suggests that there are many gaps both in terms of young people accessing healthcare services and also in having the opportunity to voice their own preferences for the HIV and contraceptive services that are available to them. Thus, exploring and understanding the preferences of young people for HIV and contraceptive services could potentially have a positive influence on critical sexual and reproductive health issues that they encounter. This study sought to explore the opinions and preferences of high school learners and stakeholders who support the provision of services to adolescents with regard to the acceptability and feasibility of various models for offering HIV and contraceptive services to learners. In addition, it sought to identify the characteristics of HIV prevention and contraceptive service delivery that are important to school-going South African adolescents when making decisions regarding whether and how to access care.

## Methods

### Study design, sampling and setting

South Africa currently has various healthcare service delivery models and this study focuses on the five key models that provide primary healthcare services in both urban and rural settings. The chosen models include primary health clinics, public-private partnerships, community health outreach, school-based services and mobile units. These models are all equipped at varied levels to deliver a variety of primary healthcare services including HIV and contraceptive healthcare services to the public sector. Findings from KIIs focused on stakeholder perceptions relating to acceptability and feasibility of these models for adolescents. FGDs focused on identifying the most important characteristics of HIV prevention and contraceptive service delivery from adolescents themselves.

Using a convenience and purposive sampling strategy, we conducted 22 in-depth stakeholder interviews (KIIs) with key informants and 8 focus group discussions (FGDs) with 55 learners between March and October 2018. The stakeholders included government officials involved in the Integrated School Health Programme (ISHP), nurses assigned to schools, school governing bodies consisting of school principals, teachers, parents of learners and other community representatives. The in-depth interviews with stakeholders was done initially using purposive sampling and then snowballing which also allowed participants within the study to suggest other possible participants. For the FGDs, the population included male and female secondary school learners, aged 15 years and older who were in grades 9–12. The research team recruited learners depending on availability of learners across all grades. FGDs were stratified by sex in order to encourage conversation and discussion.

In terms of study setting, this qualitative research was conducted with learners from three public secondary schools in Gauteng Province, South Africa within the districts of Johannesburg East, Gauteng East and Ekurhuleni North. Criteria used to select schools included: (a) secondary schools with > 1000 learners, (b) schools in districts with a high HIV prevalence among 15–24 year olds, (c) schools in wealth quintiles 1–3 and (d) schools with high rates of pregnancy. We selected schools representing low to moderate socioeconomic (SES) settings (wealth quintiles 1–3) and areas where either HIV or contraceptive outcomes (or both) are poor (as determined by HIV prevalence and an estimated teenage pregnancy rate).

### Data collection

For the KIIs, after consent forms for interviews and audio-recording were signed and returned, interviews were then conducted by a trained interviewer in English or the preferred local language and within a private venue. Each interview was conducted using a guide, took 60 to 90 minutes to complete and was audio-recorded. For the FGDs, participants were selected through their respective schools based on willingness to participate after they had received a study briefing. All participants under the age of 18 were given assent and parental consent forms, whilst those above 18 could consent of their own accord. After assent/consent forms for discussions and audio-recordings were signed and returned, FGDs were conducted at school after school hours with reimbursements being provided for transport. FGDs were audio-recorded; lasted 30 to 90 minutes and were conducted by trained facilitators either in English or preferred local language using a discussion guide.

### Data Analysis

Audio files were transcribed verbatim and translated into English if required. Data was collated using NVivo version 11, a qualitative data analysis (QDA) computer software package. Transcripts were reviewed by four researchers and interviews were coded using thematic analysis. The analysis involved multiple readings of transcripts by researchers to identify recurring themes and two master codebooks were initiated – one each for stakeholders and learners. The authors then revised versions of the codebooks according to emerging themes. The codebooks were reviewed, and themes redefined over several meetings until consensus was reached on the definition of all themes. New themes that emerged during this process were defined and added to the codebooks. Four interview and four FGD transcripts were coded by all members of the study team and compared for inter-coder reliability. Transcripts were re-coded if a new theme emerged or if a theme was redefined and consensus was reached.

### Ethical considerations

This study was approved by the Institutional Review Board of the Human Research Ethics Committee (Medical) of the University of the Witwatersrand (Protocol 170213) and the University of Boston Institutional Review Board (IRB) (Protocol H-35987). The study was also approved by Gauteng Department of Education (2017/323) and all relevant districts within Gauteng province.

## Results

Participants in both groups unanimously concurred that the barriers preventing adolescents from accessing HIV and contraceptive services that are currently at their disposal outweighed the facilitators that encouraged them to seek these out these same services.

### Characteristics of stakeholder participants and focus group discussion participants

#### Characteristics of interview participants

A total of 22 KIIs were conducted with various stakeholders from the Department of Basic Education (National, Provincial and District levels), National Department of Health (NDOH), schools, stakeholders from the community such as the school governing body, primary healthcare nurses and learner support agents who were employed as peer educators by the NDOH (Table 1).

#### - Characteristics of focus group discussion participants

A total of eight FGDs were conducted with 55 (24 males, 31 females) learners across two schools, from grades 9 to 12 (Table 2). The majority of participants were 17 years and older (55%) and were from grade 10 (53%).

### Key findings from stakeholder interviews and focus group discussions

Despite different guides being used for stakeholders and learners in this study, findings displayed a similarity between KII and FGD responses and due to this, results are presented together below:

#### • BARRIERS TO ACCESSING HIV AND CONTRACEPTIVE SERVICES

**Figure 1:**
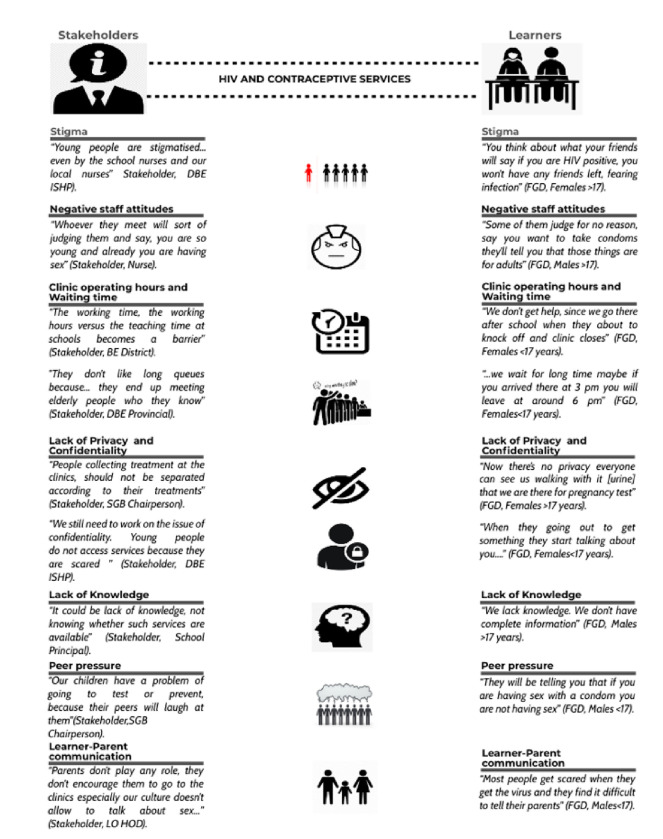
Barriers to accessing HIV and contraceptive services

The most common barriers reported to face young people accessing HIV and reproductive services are summarised in Fig. 1 and explained below.

##### - Stigma and disclosure

There is a strong lack of respect for youth seeking out HIV and reproductive healthcare services because they are deemed to have done something that society does not approve of. This stigma is noted to start at home and is further perpetuated at health care facilities by staff. Both stakeholders and learners stated that as long as young people still fear that they are going to be seen and judged by society or even their peers when they attend healthcare facilities for HIV and contraceptive services, then this perception is always going to be a significant accessibility barrier. Participants also stated that it is because of stigma that learners are afraid to discuss or confide in others about HIV and contraceptive issues. Learners also feel uncomfortable waiting in lines at facilities because they fear being seen by people who would know which families they are from and then disclose this to the broader community. It was suggested that learners in uniform be prioritised to avoid waiting and this will thus reduce the risk of accidental disclosure and hence reduce stigmatisation toward them.

##### - Unfriendly and judgemental staff attitudes

Participants indicated that nurses often communicate in a judgemental manner and are verbally abusive toward learners who come to health facilities seeking out HIV and reproductive healthcare services. Stakeholders themselves, including healthcare service providers like nurses, admitted that negative staff attitude from staff at facilities makes it challenging for learners to access HIV services and contraceptive services. It was further specified by most participants across both groups that older healthcare service providers at facilities are more likely to be unfriendly toward learners instead of advising them as younger staff were perceived to do. Learners are even noted to be often reprimanded by older staff for their being sexually active at a young age. Thus, negative staff attitude was recognised by most participants as a barrier that makes it difficult for learners to access care freely due to the unwelcoming attitude they receive from certain staff members.

##### - Clinic operating hours and waiting times

Participants stated that, because young people attend school at certain designated times, the normal standard clinic operating times are not favourable for learners to access most clinic services. Clinic operating times are not structured in a manner that accommodates the schedule of learners. Participants suggested offering flexible operating times to encourage young people to access healthcare services. Learners also reported that the long waiting times at clinics are a deterrent as they are only able to attend the clinic after school hours, when clinics are busiest and then have to spend a large amount of time waiting to be attended to at the clinics. There was also an agreement amongst most participants that there are longer queues in the healthcare facilities at the time that learners get there and there is no flexibility to prioritise learners. Hence learners are discouraged from going to these facilities when they do require assistance and advice.

##### - Lack of privacy and confidentiality

Participants indicated that nurses often conduct tests in front of other patients and staff due to the lack of private consulting rooms within many facilities. As HIV and contraceptive services for adolescents are considered to be sensitive subjects, this lack of privacy makes learners uncomfortable and discourages their return. Participants reported that patients are discriminated against by nurses based on their ailments and patients with different sicknesses are even placed in different queues thus exposing them to unintended disclosure. This issue was exacerbated in the mobile van model where learners felt uncomfortable discussing their health care needs as they perceived that they could easily be heard by other patients.

Participants pointed out that the failure to keep information that learners share with healthcare providers confidential was a significant factor that was responsible for triggering fear among the learners who attempt to access health services. Healthcare providers are deemed to be unethical with the issue of confidentiality especially when dealing with learners as they are not allowed to be sharing patient information without consent to the public at large. This is a significant barrier because even though services are available and accessible, the notion that service providers will breach confidentiality deters learners from accessing these services altogether.

##### - Lack of knowledge

Participants reported that learners lack essential knowledge about the availability of healthcare services and how to access the existing services. This gap in knowledge is filled by information obtained from unreliable sources such as peers which is prone to contain misconceptions (including those related to side-effects e.g. weight gain and long-term infertility).

##### - Peer pressure

Some participants indicated that learners do not access HIV and contraceptive services fearing that their peers would mock them which translates into a fear of seeking out contraceptive services. This however, is contradictory to what some learners say about having to rely on their friends for HIV and contraceptive information. Peer pressure was also noted as a reason for engaging in unprotected sex as sex with protection was considered by some youth to be the equivalent of not having sex at all.

##### - Learner-parent communication

It was also intimated by participants that there is a lack of communication between learners and their parents when it comes to making decisions about HIV and contraceptive services. There was the perception that learners do not get proper guidance regarding HIV and contraceptive services and rely on information from their friends that may not always be correct which leads to poor uptake of HIV and contraceptive services. Furthermore, social and cultural expectations often inhibit learners from discussing these issues with their parents.

#### • FACILITATORS TO ACCESSING HIV AND CONTRACEPTIVE SERVICES

**Figure 2:**
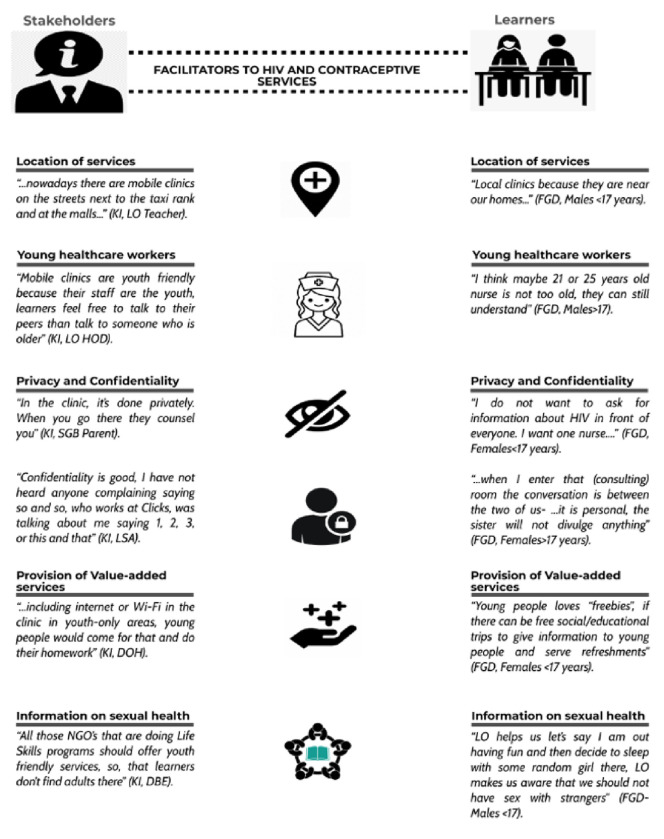
Facilitators to accessing HIV and contraceptive services

The facilitators that participants identified were all directly aligned with the barriers encountered suggesting that if these barriers are eliminated then accessibility to HIV and contraceptive services will increase. These are summarised in Fig. 2 and explained below.

##### - Location of services

Participants emphasised that services should be conveniently located to provide easy access for learners. It was reported by some participants that mobile clinics and school programs did improve access and mitigate the issues of costs and time related to long travelling distances. Participants also reported that being offered HIV and contraceptive health services at schools has meant that these services can be made to be readily available at any time during school hours for those seeking them out.

##### - Young healthcare workers

Many participants noted that learners preferred receiving services from younger healthcare providers. Younger healthcare providers were experienced as being friendlier, more approachable and understanding about the issues that young people face and as a result are perceived to be less judgemental. The judgemental and negative attitude from older staff impacted learner’s experiences at healthcare facilities resulting in them not being willing to return to care. Older healthcare workers are also perceived to be less knowledgeable about current healthcare practices.

##### - Privacy and confidentiality

Participants reported that ensuring privacy in healthcare facilities encourages learners to access healthcare services more freely as there is less fear of being seen by people they know or of their information being divulged in front of other staff and patients. Participants from FGDs (learners) worried about patient-provider confidentiality as many of the providers they encountered at healthcare facilities were members of their communities.

##### - Provision of value-added services

Many participants were supportive of including entertainment to improve the reach and effectiveness of current adolescent health programs aimed at HIV and contraceptive services provision. This includes the provision of live music entertainment as well as live shows to attract youth to these facilities. In addition, the inclusion of a range of value-added services like free Wi-Fi, youth-only waiting areas and food were also thought likely to appeal to adolescents and encourage them to access these healthcare services. Value added services may also encourage learners to invite their peers to attend these healthcare facilities with them, thus enabling a wider reach to learners.

##### - Information on sexual health

Many participants emphasized that Life Orientation subject sessions at school should continue to educate learners about HIV and contraceptive services. It was suggested that non-governmental organisations (NGOs) should assist with educating youth by presenting subjects around sexual health at schools. NGOs are perceived as neutral providers of sexual health information and services that target youth specifically and are thus seemingly well placed to impart this educational information. They also have the capacity to create a youth-friendly environment by employing young adults which works to increase learner comfort for accessing these NGO services. In terms of educating youth, group settings and community involvement were noted to be successful endeavours. In particular, expos, adherence clubs and churches are noted to be well placed for sharing information with youth as well as for engaging young people living with HIV (PLWH) to help relay messages about HIV and sexual health to youth.

## Discussion

Despite different questions being posed to stakeholders and learners in this study, the resulting data produced various overlapping factors between both groups of participants in terms of the most critical barriers and facilitators that youth encounter when accessing HIV and contraceptive services. Findings conveyed a strong relationship between the gaps in delivery of HIV and contraceptive services and the preferences of learners for accessing HIV and contraceptive services. The key point of note is that similar to other study findings[21],[22], we also noted in this study that accessibility barriers and facilitators fall across a spectrum of factors (including community, facility and individual personal level attributes) that further influences how these may be addressed by healthcare policy decision-makers.

Stigma as a cross-cutting factor, was noted in this study to be one of the barriers most likely to deter youth from accessing HIV and contraceptive healthcare services. Stigma has become so ingrained within the very fabric of society at a community level, that youth are even questioned by facility security guards about their purpose of the visit. Since many youths have never accessed healthcare services alone before, trying to navigate this new territory is further impeded by these challenges that they are not prepared for. This includes being further stigmatised by healthcare staff who openly question youth about whether their sexual activities are age appropriate. Stigma is also noted by numerous studies to be a universal issue affecting individuals in all areas of their lives and is shown to affect healthcare-seeking behaviour [23], [24]. Context-specific innovative programs, example mastery experiences through sport which will also include vicarious experiences whereby youth observe peers succeeding at certain sports, are needed to build up self-efficacy and resilience within youth themselves which could then empower them to deal with experiences of stigma more effectively.

On a facility-level there are numerous critical factors that relate both to services rendered in terms of negative staff interactions as well as services affected by facility operational and structural issues. In summary and as evidenced in previous literature [25], [26], youth in this study also felt that they had to wait too long to be consulted, had to miss school due to inconvenient operating hours and that they were being verbally abused, judged and intimidated by older staff who reprimanded them openly. The lack of private consulting spaces within these facilities further impacted on the discretion of services offered as well as interactions between staff and patients and this has been noted to be common across other healthcare settings too [27], [28]. Logistically, a lack of privacy is linked to staff grouping same-condition patients together to get through queues quickly, older healthcare staff in attendance due to limited vacancies and budget to employ younger staff, which has also been shown to ultimately lead to overburdened staff who then have low tolerance levels for youth [29], [30]. This in turn creates a dilemma for youth accessing these services which is also further perpetuated by experiences of staff breaching confidentiality and sharing their personal information out of the facility and into the very communities that these youths are part [30], [31]. Thus, addressing these critical issues through novel adjustments or adapting the flow of existing facilities and accompanying these with workshops training staff to be more confidential and welcoming [32], [33]; may be a crucial link in improving uptake of services by learners.

On an individual level, the lack of effective communication around HIV and contraceptive issues between young people and their parents indicates a possible lack of interventions specifically targeting parents of young sexually-active learners. A point of note is the notion that when young people feel free and confident or trusting enough to open up and communicate about their sexual issues to those they are closest to, they will also be confident, open and free to go out and easily access services that they need without any fear or confusion. Participants who emphasized a lack of knowledge on where to access these services also indicated that they do not receive this information at school during the subject session designated to offer this type of health information to learners. It has been shown that learners are unlikely to return to care due to such barriers and the opportunity to further educate them about risky behaviour will be lost which in turn may lead to further cross-infections amongst youth as noted by other studies [34], [35]. There is also evidence pointing to the potential risk of misconceptions spreading since learners rely on each other for information; as well as possibly future anxiety about returning to care even after these challenges are potentially addressed [36], [37].

Despite the multitude of barriers, participants noted that if value-added services are offered to them (e.g. free food and WI-FI) and, youth-friendly waiting areas are created then these will serve as facilitators for young people to access HIV and contraceptive services more readily. Learners will also now have the ability to do school homework while they are waiting due to availability of internet resources. It was further suggested that non-governmental-organisations should be invited to schools to provide health information to learners which is an area that warrants more research. A premise is that all these facilitators hold promise for future interventions but further studies looking into the preferences of young people weighed up against what is feasible are needed.

The findings established in this paper are in line with the literature and confirm that young people need to be involved in order to inform changes to existing health care systems in South Africa [38]. Learning about their preferences from youth themselves will enable key decision-makers to effect interventions that speak directly to the needs of this population within our current healthcare system. Thus, a research methodology such as a discrete choice experiment which is a study design used to elicit individuals’ preferences for different goods or services that is being increasingly employed in health economics has been conducted as a follow-up to this study in order for policy recommendations to be provided to decision-makers [39].

## Limitations of the study

Given that saturation was reached in the focus group discussions and key informant interviews it is possible that these barriers and facilitators would be identified by learners in other schools but it is also possible that additional or different barriers and facilitators would be identified at other schools or in other provinces. The selected schools were classified as serving a community from wealth quintiles 1 and 2 and so schools in wealthier communities could present with different preferences. However, it must be noted that quintiles were not always a reasonable indicator of socio-economic status given that less funding goes to schools in higher quintiles and this is accompanied by the additional challenges of independently being able to access care among those learners whose healthcare access was linked to their parent’s medical aid. The study design was also limited in terms of school selection in that eventually only schools that were willing to participate, thus this excluded those schools who were not willing for us to discuss contraceptive and HIV service access with their students and so may have excluded those learners who had even greater challenges in gaining access to these services.

## Conclusion

Stakeholders and learners shared similar concerns regarding the barriers to HIV and contraceptive services for school going adolescents. Unless these barriers are addressed and resolved, many learners will continue to fall through the gaps and expose themselves further to additional health risks. This does not just include their access to health services but also the confidence and ability to navigate public health services when needed, and the resilience needed to overcome difficult issues like stigma and judgemental attitudes which they have no control over. The provision of private, confidential youth-friendly services at locations and times that can be easily accessed by learners was found to be key in this study. It is clear that health care service delivery to this population needs to be differentiated from the current standard service delivery approaches that are adult centric and do not take into account the unique needs or preferences of school going adolescents.

## Figures and Tables

**Table 1.** Stakeholders – Occupation

	N = 22 (%)

**Occupation**	
DBE	6 (27.27)
DOH	4 (18.18)
School Principal	1 (4.55)
LO (Life Orientation) Teachers/LO HOD (Head of Department)	4 (18.18)
SGB (School Governing Body) Parents	3 (13.64)
SGB Teachers	1 (4.55)
SGB Chairpersons	3 (13.64)
**Gender**	
Males	73 (31.82)
Females	15 (68.18)

**Table 2.** Focus Group Discussions

Sites	n = 55 (%)

**Site 1**	31 (25.00)
**Site 2**	24 (25.00)
**Age at time of interview**	
15 to 16 years	25/55 (43.45)
17 years and older	30/55 (54.55)
**Gender**	
Males	24 (43.64)
Females	31 (56.36)
**Class Grades**	
Grade 9	9 (16.36)
Grade 10	29 (52.73)
Grade 11	4 (7.27)
Grade 12	13 (23.64)

## Data Availability

The data that support the findings of this study are available from the corresponding author upon reasonable request.

## Abbreviations

FGD: Focus Group Discussion
KII: Key Informant Interviews
HIV: Human Immunodeficiency Virus
SSA: Sub-Saharan Africa
SGB: School Governing Body
ISHP: Integrated School Health Programme
STI: Sexual Transmitted Infection
QDA: Qualitative Data Analysis
IRB: Institutional Review Board
NDOH: National Department of Health
DBE: Department of Basic of Education
LO: Life Orientation
HOD: Head of Department
NGO: Non-Government Organisation
PLWH: People Living with HIV
